# “Sarcopenia and intramuscular fat deposition are associated with poor survival in Indonesian patients with hepatocellular carcinoma: a retrospective study”

**DOI:** 10.1186/s12876-019-1152-4

**Published:** 2019-12-30

**Authors:** Yan Mardian, Yoshihiko Yano, Neneng Ratnasari, Lina Choridah, Widya Wasityastuti, Nurhuda Hendra Setyawan, Yoshitake Hayashi

**Affiliations:** 1 Division of Infectious Disease Pathology, Department of Microbiology and Infectious Disease, Kobe University Graduate School of Medicine, Kobe, Japan; 2 Division of Gastroenterology, Department of Internal Medicine, Kobe University Graduate School of Medicine, Kobe, Japan; 3 Division of Gastroenterohepatology, Department of Internal Medicine, Dr. Sardjito Hospital, Faculty of Medicine, Gadjah Mada University, Yogyakarta, Indonesia; 4 Department of Radiology, Dr. Sardjito Hospital, Faculty of Medicine, Gadjah Mada University, Yogyakarta, Indonesia; 5 Division of Molecular Medicine & Medical Genetics, Department of Pathology, Kobe University Graduate School of Medicine, Kobe, Japan

**Keywords:** Sarcopenia, IMF deposition, Hepatocellular carcinoma, Prognosis, Indonesia

## Abstract

**Background:**

A large-scale Japanese study showed that low skeletal muscle index (SMI) and intramuscular fat (IMF) deposition are associated with hepatocellular carcinoma (HCC) survival. Here, we evaluated the effects of SMI and IMF on the survival of Indonesian HCC patients, whose characteristics differ from those of Japanese patients.

**Methods:**

SMI and mean muscle attenuation (MA) were evaluated using computed tomography images of the third lumbar vertebra (L3) in a prospective cohort of 100 Indonesian HCC patients. Clinical, laboratory and body composition data were analysed using the Kaplan–Meier method and Cox regression model to investigate which factors are associated with prognosis.

**Results:**

Of 100 patients, 31 were diagnosed with sarcopenia (L3 SMI value ≤36.2 cm^2^/m^2^ for men and ≤ 29.6 cm^2^/m^2^ for women), and 65 had IMF deposition (MA value ≤44.4 HU for men and ≤ 39.3 HU for women). These groups had shorter median survival than the reference groups (both *P* < 0.0001). In multivariable analysis, sarcopenia (hazard ratio [HR], 1.921; *P* = 0.016), IMF deposition (HR, 3.580; *P* < 0.001), Barcelona Clinic Liver Cancer (BCLC) stages C and D (HR: 2.396, *P* < 0.01 and HR: 6.131, *P* < 0.01, respectively), Japan Integrated Staging (JIS) score 4 (HR: 2.067, *P* = 0.020), and male gender (HR: 3.211, *P* < 0.001) were independently associated with mortality.

**Conclusion:**

Sarcopenia and IMF deposition showed superior value in combination with BCLC stage and JIS score for predicting the survival of Indonesian HCC patients. Increased awareness and strategies to prevent or reverse these factors might improve patient outcomes. (Electric word counts: 249).

## Background

Hepatocellular carcinoma (HCC), a primary malignancy derived from hepatocytes, accounts for approximately 80% of all liver cancers with approximately 700,000 new cases of HCC are diagnosed each year globally [1]. The prognosis of the patients with HCC is very poor which places it as the second leading cause of cancer-related death worldwide [1, 2]. The incidence of HCC is rising in developed countries and continues to be high in endemic hepatitis B areas, such as Asia-Pacific region [3].

In Indonesia, liver cancer is the fifth most commonly diagnosed cancer and was the fourth leading cause of cancer-related deaths in 2012 [4]. Because of its endemicity of hepatitis B virus (HBV) infection, which is the third-highest prevalence worldwide, HBV is regarded as the leading cause of HCC in Indonesia [5]. A previous study performed in Indonesia demonstrated that most patients are diagnosed at intermediate to advanced stages (Barcelona Clinic Liver Cancer [BCLC] stage B or C), are relatively young (mean age, 54 years), and have a poor prognosis with a median overall survival of 138 days [6]. However, patients with advanced HCC at diagnosis are ineligible for curative therapy and generally have poor long-term outcomes [7].

Despite its high mortality rate, the prognostic factors for HCC remain controversial because the long-term prognosis of HCC is strongly associated with hepatic functional reserve and the stage of cancer progression [8]. Previous studies demonstrated that changes in body composition are associated with poor outcomes in cancer patients including HCC [9]. A large-scale retrospective study conducted in Japan revealed that loss of skeletal muscle, known as sarcopenia, and intramuscular fat (IMF) deposition, measured by computed tomography (CT), are related to the prognosis of HCC [2]. The pathophysiological mechanism linking sarcopenia and IMF deposition may include insulin resistance and increased levels of inflammatory cytokines, which are also associated with the progression of HCC [2, 10].

However, the role of body composition in Indonesian HCC patients, whose clinical status differs from that of Japanese patients, in terms of the age of onset, causative virus, and stage at diagnosis [2]; still remains unclear. In this study, we evaluated the impact of sarcopenia and IMF deposition on the survival of Indonesian HCC patients and identified the key prognostic factors for HCC in these patients.

## Methods

### Study design, patient selection, and follow-up strategy

The patients were enrolled at the Dr. Sardjito Hospital, Gadjah Mada University, Indonesia, a tertiary centre, between 2016 and 2018. This study was a prospective cohort study that included patients with HCC (confirmed by clinical judgment, dynamic CT, and/or fine needle aspiration biopsy) aged > 18 years. Patients with poorly controlled ascites were excluded because this might lead to overestimation of body mass index (BMI). Patients diagnosed with other malignancies up to 5 years before the HCC diagnosis were also excluded.

Patients underwent an abdominal CT scan within 1 month before or soon after the first admission. All patients signed a written informed consent form confirming their willingness to participate in the research. The authors collected data on the participants, including demographics and history of the present illness as well as past illnesses and family history, performed physical and laboratory evaluations, and performed the abdominal CT scan examination, which was validated by anatomical radiologists.

Liver-related morbidity and mortality rates were determined monthly from the date of the first admission for HCC until the date of death/or the end of the study (March 2019), whichever came first. Clinical, laboratory, and body composition assessments were comprehensively analysed using the Kaplan-Meier method and Cox regression model to investigate the critical features associated with prognosis.

### Study measures

Clinical characteristics included in the study were age at enrolment, sex, BMI, presence of comorbidities (diabetes, hypertension), aetiologies of liver disease, alcohol consumption, history of cardiovascular or cerebrovascular disease, history of previous HCC treatment, presence of ascites and hepatic encephalopathy, and level of functioning, as measured by the Eastern Cooperative Oncology Group (ECOG) performance status scale. Laboratory characteristics included liver function tests (aspartate aminotransferase [AST] and alanine transaminase levels), total bilirubin, albumin levels, creatinine, electrolytes, complete blood count, C-reactive protein (CRP), international normalised ratio (INR), and alpha-fetoprotein (AFP) levels.

Tumour features were assessed using baseline CT imaging and included tumour size, number, type, presence of vascular invasion, direct invasion to adjacent organs and/or perforation of the visceral peritoneum, regional lymph node metastasis and distant metastasis. Child–Turcotte–Pugh (CP) stage score was calculated at baseline to evaluate liver function. BCLC stage, the Liver Cancer Study Group of Japan (LCSGJ) tumour node metastasis (TNM) stage, American Joint Committee on Cancer (AJCC) TNM stage 8th edition, and the Japan Integrated Staging (JIS) score, which is calculated by adding the TNM LCSGJ stage and CP score, were used in this study as the staging systems of HCC.

Body composition components, including skeletal muscle index (SMI) and muscle attenuation (MA), were measured on transverse CT images at the third lumbar vertebra (L3). Images were analysed using SliceOmatic V5.0 (Tomovision, QC Canada), which enables specific tissue demarcation using Hounsfield unit (HU) thresholds. A tissue threshold of − 29 to + 150 HU was used to determine the skeletal muscle area, which included the psoas, erector spinae, quadratus lumborum, transversus abdominis, external and internal obliques, and rectus abdominis muscles.^2^ The skeletal muscle area was normalised to height in meters squared and expressed as SMI in cm^2^/m^2^. Mean MA was calculated using the same CT images to assess skeletal muscle quality and lipid content.

Sarcopenia was defined as a L3 SMI value ≤36.2 cm^2^/m^2^ for men and ≤ 29.6 cm^2^/m^2^ for women, as previously described^2^. Low MA is defined as mean muscle attenuation ≤44.4 HU in men and ≤ 39.3 HU in women [2]. A low MA value indicates an IMF content contributing to muscle weakness independent of age-associated loss in muscle mass. The CT scan results were interpreted by two expert radiologists who underwent inter-observer variability testing using the Kappa Score and showed a reliability > 75%.

### Statistical analysis

Survival was calculated using the Kaplan–Meier method and compared using the log-rank (Mantel–Cox) test. GraphPad Prism, version 7 (GraphPad Software, La Jolla, CA, USA) was used to generate survival curves. Univariate and multivariate analyses were performed to identify factors independently associated with liver-related mortality. Univariate and multivariate analyses of overall survival were performed using Cox regression models, and the results are presented as hazard ratios (HR) with 95% confidence intervals (CI). *P* values were derived using the Wald test. Variables exhibiting significant associations in univariate analyses were included in multivariate analyses. Differences in categorical variables and continuous variables were compared using the Pearson χ^2^ test and Student’s *t*-test, respectively. Statistical analyses were performed using SPSS version 22 (IBM Corporation, Armonk, NY, USA). All *p*-values were two sided, and the level of significance was set at *P* < 0.05.

## Results

### Patient characteristics

A total of 124 consecutive patients with HCC admitted to Dr. Sardjito Hospital were identified during the study period. Twenty-four patients were excluded because CT scan imaging data and/or required clinical/laboratory data were missing or because of concomitant malignancies. Therefore, 100 HCC patients were included in the final analyses. There were 74 men (74%), and the mean ± standard deviation age was 55.03 ± 11.20 years. The most common cause of HCC was hepatitis B infection (58%). Non-B non-C type (NBNC) HCC, which tests negative for HBV and HCV markers, ranked second (34%).

All enrolled patients initially presented to the hospital after the onset of symptoms. The most common clinical signs or symptoms were malaise and nausea (76%), abdominal pain (68%), weight loss (80%), and hepatomegaly (92%); jaundice, ascites, splenomegaly, and hematemesis/melena were observed in 25, 32, 30, and 11% of patients, respectively. The BCLC stage distribution and JIS score data suggested that most the patients were diagnosed at an intermediate to advanced stage (BCLC stage C, 59%; JIS score 3, 31%) and had relatively large tumours (mean diameter, 12.05 ± 4.97 cm). Interpretation of the CT scan indicated that the most common tumour types in Indonesian HCC patients were multiple (70%) and nodular (65%) types.

Because of the advanced-stage presentation, all patients were given only palliative (transarterial chemoembolisation [TACE]) or supportive treatment (end-stage life support). Some patients did not undergo TACE because of the long waiting list or died while waiting for TACE. Treatment with radiofrequency ablation (RFA) and the use of sorafenib were limited because of their high costs and lack of coverage by the national health insurance. The rate of alcohol consumption was low (only 8%) among these patients presumably because of religious practices and the limited availability of alcohol in the public market. Complete baseline characteristics are summarised in Table 1.

**Table 1:** Baseline characteristics of the study population

Characteristic	*N* = 100
Age, mean ± SD	55.03 ± 11.20
Gender, male, %	74
Body mass index (kg/m2), median (IQR)	18.83 (17.78–23.11)
< 18.5 (underweight), N (%)	31
18.5–22.9 (normal weight), N (%)	44
23.0–24.9 (overweight), N (%)	6
≥ 25.0 (obesity), N (%)	19
Viral status, %	
HBV/HCV/HBV + HCV/none	58/8/0/34
Child–Pugh classification, %	
A/B/C	60/32/8
BCLC stage, %	
A/B/C/D	7/25/59/9
ECOG PS, %	
0/1/2/3	32/41/22/5
LCSGJ TNM stage, %	
1/2/3/4	0/21/32/47
AJCC TNM stage 8th edition, %	
1/2/3/4	20/5/32/43
JIS score, %	
1/2/3/4/5	18/23/31/23/5
Treatment, %	
Curative (liver transplant, resection, PEIT, RFA)	0
Palliative (TACE, sorafenib)	65
Supportive (end-stage life support)	35
Alcohol consumption, %	
Yes/no	8/72
Clinical signs and symptoms, %	
Malaise, nausea, vomiting/hematemesis or melena	76/11
Abdominal pain/weight loss	68/80
Jaundice/ascites	25/32
Hepatomegaly/splenomegaly	92/30
Tumour diameter, mean ± SD (cm)	12.05 ± 4.97
Type of tumour, %	
Nodular/diffuse	65/35
Single/multiple	30/70

*SD* Standard deviation, *BCLC* Barcelona Clinic Liver Cancer, *ECOG PS* Eastern Cooperative Oncology Group Performance Status, *TNM* Tumour-node-metastasis, *LCSGJ* Liver Cancer Study Group of Japan, *TNM* Classification system, *AJCC* American Joint Committee of on Cancer, *JIS* Japan Integrated Staging, *PEIT* Percutaneous ethanol injection therapy, *RFA* Radiofrequency ablation, *TACE* Transarterial chemoembolisation

### Association between sarcopenia and HCC survival

Of the 100 patients included in the final analysis, 31 were diagnosed with sarcopenia (L3 SMI value ≤36.2 cm^2^/m^2^ for men and ≤ 29.6 cm^2^/m^2^ for women), of which 23 (75%) were men. At baseline, the median L3 SMI value was 39.94 ± 8.03 cm^2^/m^2^ in men and 32.53 ± 5.23 cm^2^/m^2^ in women. Inter-observer concordance on the L3 SMI value and mean muscle attenuation was > 95%.

**Fig. 1:**
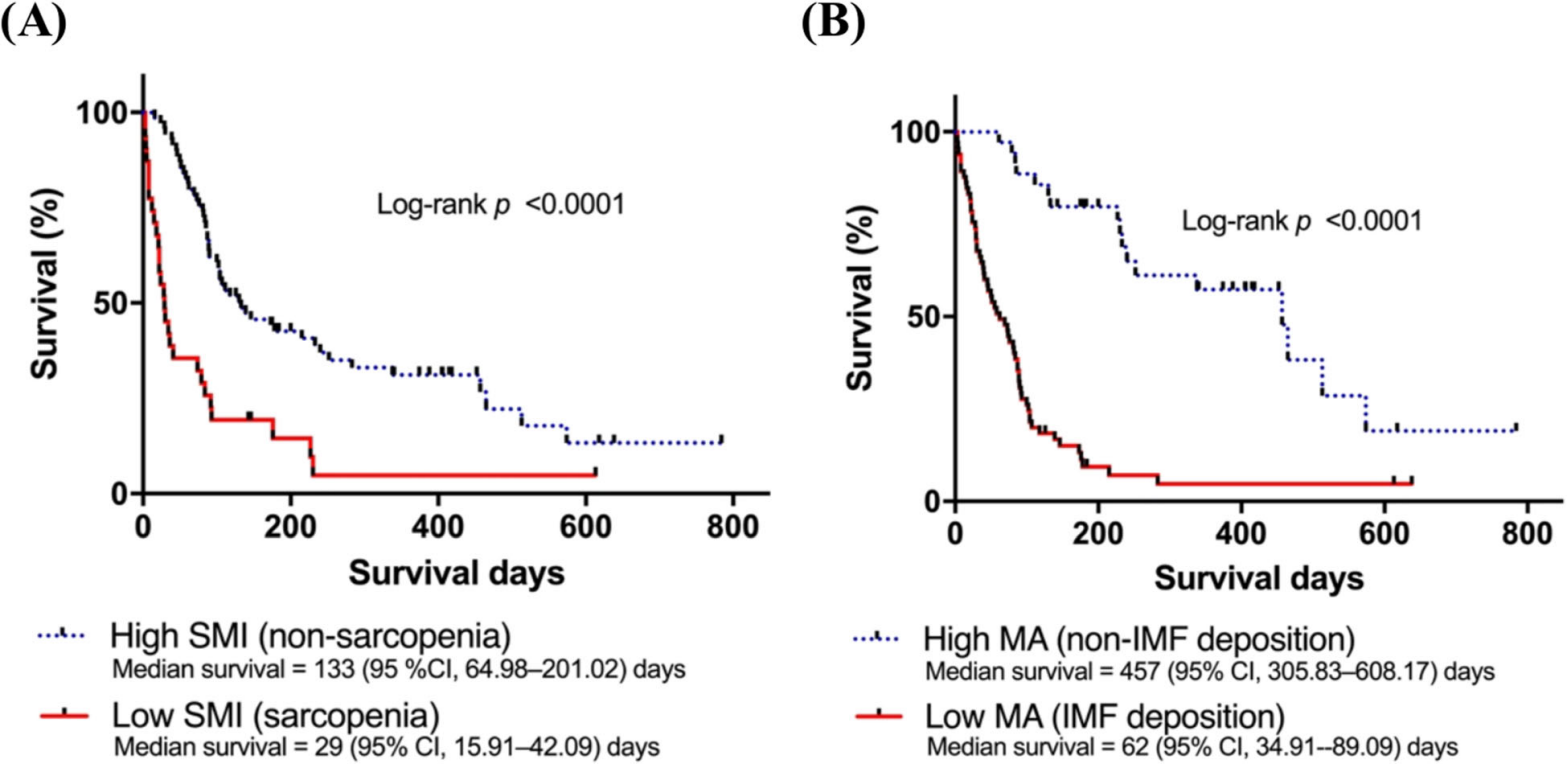
Impact of body composition on overall survival curves. Low SMI (**a**) and IMF deposition (**b**) were significantly associated with lower survival of Indonesian HCC patients. Kaplan–Meier method was used to generate survival curves. CI, confidence interval; IMF, intramuscular fat; MA, muscle attenuation; SMI, skeletal muscle index

The median survival of HCC patients was 92 ± 8.5 days. The Kaplan–Meier curves showed that median survival was shorter in patients with sarcopenia than in patients with high SMI/non-sarcopenia (29.0 ± 6.68 versus 133 ± 34.70 days, *P* < 0.0001), as shown in Fig. 1. The Cox regression model indicated that sarcopenia was associated with poor prognosis, with a mean 3-month overall survival rate of 26 ± 8%, and a poor prognosis (HR, 1.921; 95% CI, 1.129–3.268; *P* = 0.016) in multivariate analysis (Table 2).

**Table 2:** Univariate and multivariate Cox regression analyses of liver-related mortality

Variable	No. at risk^a^	No. of events	3-month survival, % ± SD	Univariate Cox regression analysis			Multivariate Cox regression analysis		
				HR	95% CI	*P*-value	HR	95% CI	*P*-value
Age									
< 55 years old	47	33	57 ± 7	1.000	(reference)				
≥ 55 years old	53	44	45 ± 7	1.269	0.802–2.007	0.307			
Gender									
Male^§^	74	60	46 ± 6	1.845	1.066–3.194	0.029	3.211	1.709–6.034	< 0.001
Female	26	17	65 ± 9	1.000	(reference)		1.000	(reference)	
Body mass index									
Underweight	31	24	48 ± 9	1.108	0.658–1.865	0.700			
Normal weight	44	35	50 ± 8	1.000	(reference)				
Overweight	25	18	55 ± 10	0.796	0.468–1.468	0.519			
Sarcopenia									
No	69	49	62 ± 6	1.000	(reference)		1.000	(reference)	
Yes^c^	31	28	26 ± 8	2.950	1.836–4.740	<.0.001	1.921	1.129–3.268	0.016
IMF deposition									
No	35	17	89 ± 5	1.000	(reference)		1.000	(reference)	
Yes^c^	65	60	31 ± 6	5.487	3.106–9.693	<.0.001	3.580	1.895–6.764	< 0.001
Visceral adiposity									
No	71	55	48 ± 6	1.000	(reference)				
Yes	29	22	59 ± 6	0.890	0.542–1.461	0.644			
Viral status									
HBV	58	51	40 ± 6	2.326	1.380–3.920	0.002	1.223	0.708–2.114	0.471
HCV	8	6	50 ± 18	1.338	0.536–3.340	0.533			
None	34	20	71 ± 8	1.000	(reference)		1.000	(reference)	
Child-Pugh class									
A	60	42	68 ± 6	1.000	(reference)		1.000	(reference)	
B	32	27	28 ± 8	2.129	1.306–3.469	0.002	0.406	0.087–1.901	0.252
C	8	8	13 ± 12	5.069	2.342–10.973	<.0.001	0.578	0.110–3.031	0.516
TNM-AJCC stage AJCC - 8th Edition									
TNM-AJCC 1	20	13	85 ± 8	1.000	(reference)		1.000	(reference)	
TNM-AJCC 2	5	5	60 ± 22	3.586	1.244–10.339	0.018	1.304	0.439–3.875	0.633
TNM-AJCC 3	32	21	56 ± 9	1.618	0.797–3.286	0.183			
TNM-AJCC 4	43	38	30 ± 7	3.663	1.908–7.031	<.0.001	1.177	0.341–4.063	0.796
TNM-LCSGJ stage									
TNM-LCSSGJ 2	21	14	86 ± 8	1.000	(reference)		1.000	(reference)	
TNM-LCSSGJ 3	32	22	53 ± 9	1.687	0.850–3.347	0.135			
TNM-LCSSGJ 4	47	41	34 ± 7	3.308	1.763–6.206	<.0.001	0.835	0.453–1.542	0.565
JIS score									
JIS score 1	18	12	89 ± 7	1.000	(reference)		1.000	(reference)	
JIS score 2	23	15	61 ± 10	1.426	0.737–4.494	0.362			
JIS score 3	31	23	54 ± 9	2.128	0.818–4.409	0.038	1.642	0.679–3.974	0.271
JIS score 4^c^	23	22	17 ± 8	4.802	2.053–6.205	< 0.001	2.067	1.119–3.817	0.020
JIS score 5	5	5	0 ± 0	28.373	8.802–91.456	<.0.001	3.843	0.938–15.735	0.061
BCLC stage									
A	7	2	100 ± 0	1.000	(reference)		1.000	(reference)	
B	25	13	88 ± 7	3.817	0.829–17.584	0.086			
C^§^	59	53	37 ± 6	12.419	2.907–53.067	0.001	2.396	1.248–4.600	0.009
D^§^	9	9	0 ± 0	65.958	12.94–336.25	< 0.001	6.131	1.835–20.478	0.003
AFP level									
< 200 ng/ml	48	33	62 ± 7	1.000	(reference)		1.000	(reference)	
≥ 200 ng/ml	52	44	40 ± 7	1.916	1.210–3.034	0.006	1.063	0.636–1.777	0.815

*HR* Hazard ratio, *CI* Confidence interval, *IMF* Intramuscular fat, *HCV* Hepatitis C virus, *HBV* Hepatitis B virus, *BCLC* Barcelona Clinic Liver Cancer, *TNM* Tumour–node–metastasis, *AJCC* American Joint Committee of on Cancer, *LCSGJ* Liver Cancer Study Group of Japan, *JIS* Japan Integrated Staging; *AFP* Alpha-fetoprotein. ^a^Equal to the total number of patients (*N* = 100). ^**b**^Number of deaths during the 3-month observation period. ^c^Variables that independently predicted the HCC outcome based on univariate and multivariate analyses

**Fig. 2:**
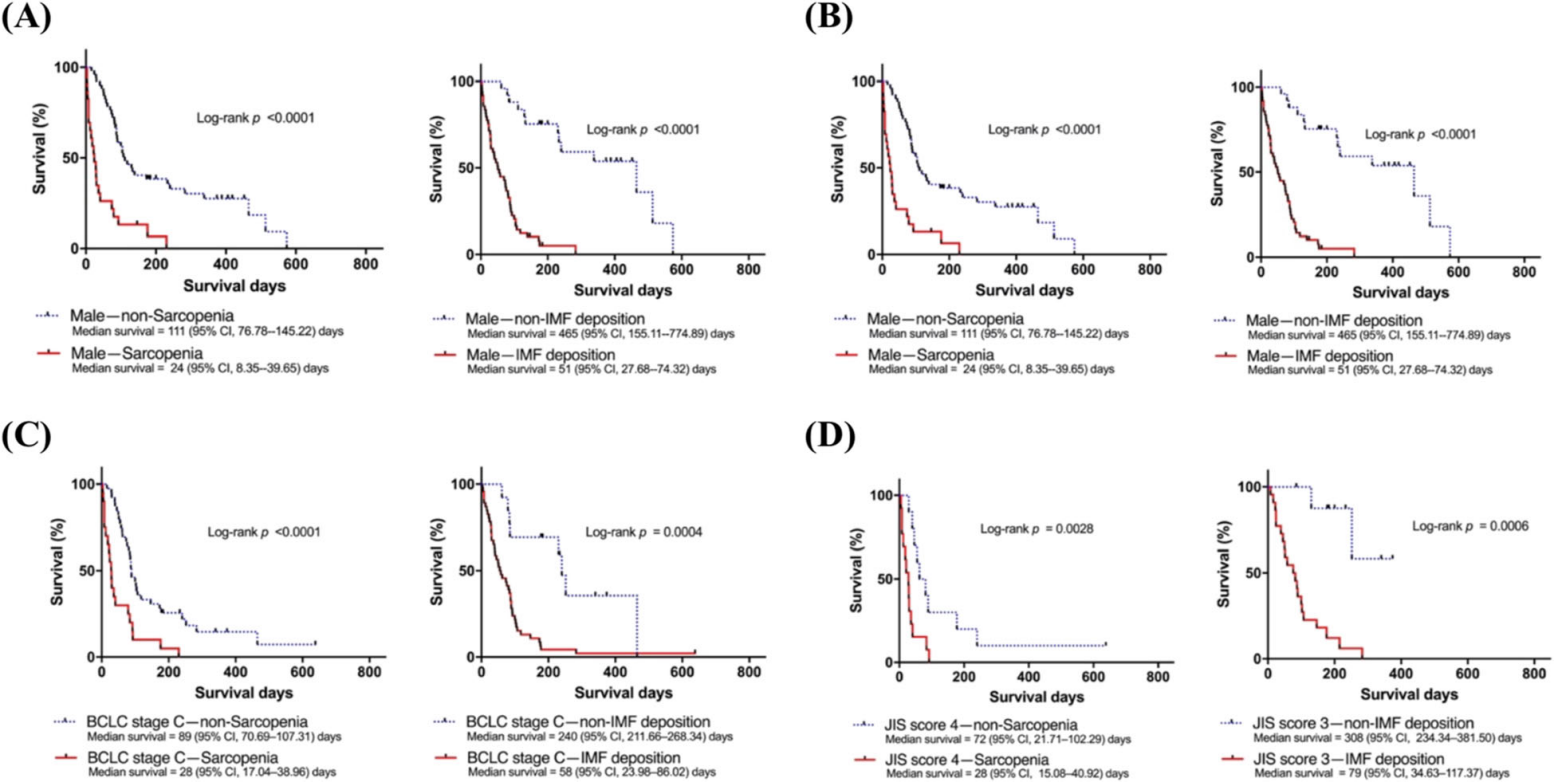
(**a**) Sarcopenia and IMF were significantly associated with worse survival in male HCC patients. (**b**) Sarcopenia and IMF were significantly associated with worse survival in HCC patients grouped by age. (**c**) Low SMI and IMF were significantly associated with poor survival in patients with BCLC stage C HCC (advanced stage). (**d**) Sarcopenia and IMF were significantly associated with poor survival in patients with JIS score 4 or 3 HCC. BCLC, Barcelona Clinic Liver Cancer; CI, confidence interval; IMF, intramuscular fat; JIS, Japan Integrated Staging; MA, muscle attenuation; SMI, skeletal muscle index; y.o., years old

To examine the effects of sarcopenia on the prognosis of HCC patients, subgroup analyses were performed to compare survival between sarcopenia and non-sarcopenia patients grouped by gender, age, BCLC stage, and JIS score (Fig. 2). Sarcopenia remained an independent predictor of reduced survival in the following subgroups: men, patients aged ≥55 years, patients with BCLC stage C, and patients with JIS score 4 (log-rank *P* < 0.0001, *P* < 0.0001, *P* < 0.0001, and *P* = 0.0028, respectively).

Baseline characteristics of the study population according to sarcopenic status are shown in Table 3. Sarcopenia was significantly associated with higher CP score, INR, and blood potassium level (*P* < 0.001, *P* = 0.037, and *P* = 0.035, respectively), whereas albumin levels were lower in patients with sarcopenia than in patients without sarcopenia (*P* = 0.010).

**Table 3:** Clinicopathological characteristics of patients with sarcopenia and intramuscular fat deposition

Variable	Sarcopenia			Intramuscular fat deposition		
	Yes (*N* = 31)	No (*N* = 69)	*P-*value	Yes (*N* = 65)	No (*N* = 35)	*P*-value
Age, mean ± SD^‡^	57.64 ± 10.65	53.85 ± 11.32	0.118	56.83 ± 10.83	51.68 ± 11.26	0.041
Gender male, N (%)	23 (74.19)	51 (73.91)	0.976	49 (75.38)	25 (71.43)	0.667
Body mass index (kg/m^2^), median (IQR)^†^	17.30 (16.11–19.85)	20.32 (19.20–24.13)	< 0.001	19.82 (17.63–23.04)	19.83 (17.85–23.12)	0.903
Viral status, N (%)			0.415			0.166
HBV	21 (67.74)	37 (53.62)		42 (64.62)	16 (45.71)	
HCV	2 (6.45)	6 (8.70)		5 (7.69)	3 (8.57)	
HBV + HCV	0	0		0	0	
None	8 (25.81)	26 (37.68)		18 (27.69)	16 (45.71)	
Child–Pugh class, N (%)^†‡^			< 0.001			< 0.001
A	10 (32.26)	50 (72.46)		29 (44.62)	31 (88.57)	
B	15 (48.39)	17 (24.64)		29 (44.62)	3 (8.57)	
C	6 (19.35)	2 (2.90)		7 (10.77)	1 (2.86)	
AST (IU/L), median (IQR) ^‡^	190.0 (85.0–262.5)	94.0 (47.0–174.0)	0.274	138.0 (71.0–256.0)	67.0 (36.0–132.5)	0.031
ALT (IU/L), median (IQR)	61.0 (27.5–82.5)	40.0 (24.0–77.0)	0.965	46.0 (28.0–82.0)	33.0 (21.5–69.0)	0.833
Total Bilirubin (mg/dl), median (IQR)	1.11 (0.68–2.25)	0.92 (0.5–1.31)	0.132	1.16 (0.55–1.80)	0.80 (0.47–1.09)	0.093
Albumin (g/dl), median (IQR) ^ab^	3.06 (2.64–3.48)	3.41 (2.99–3.91)	0.010	3.13 (2.68–3.60)	3.55 (3.22–3.94)	0.004
INR, median (IQR) ^ab^	1.19 (1.08–1.33)	1.10 (0.99–1.25)	0.037	1.17 (1.07–1.32)	1.05 (0.99–1.17)	0.001
Platelet count (× 1000/μl), median (IQR)	270.0 (194.0–297.25)	276.0 (201.0–328.0)	0.474	270.0 (194.0–317.0)	283.0 (220.5–321.0)	0.292
CRP, mean (mg/L) ± SD^b^	59.56 ± 7.12	54.89 ± 6.55	0.827	71.81 ± 7.50	34.61 ± 4.67	0.049
Creatinine, mean (mg/dl) ± SD	0.98 ± 0.53	0.88 ± 0.36	0.251	0.94 ± 0,45	0.85 ± 0.35	0.309
Random blood glucose, mean (mg/dl) ± SD	117.27 ± 39.35	121.00 ± 37.97	0.661	120.65 ± 42.29	118.19 ± 29.21	0.768
Blood sodium, mean (mEq/L) ± SD^ab^	133.61 ± 5.19	135.35 ± 5.07	0.131	133.66 ± 4.72	137.00 ± 5.24	0.002
Blood potassium, mean (mEq/L) ± SD^a^	3.97 ± 1.47	3.21 ± 1.79	0.035	3.56 ± 1.64	3.20 ± 1.88	0.334
BCLC stage, N (%)^b^			0.002			< 0.001
A	1 (3.22)	6 (8.70)		1 (1.54)	6 (17.14)	
B	3 (9.68)	22 (31.88)		9 (13.85)	16 (45.71)	
C	20 (64.52)	39 (56.52)		46 (70.77)	13 (37.14)	
D	7 (22.58)	2 (2.90)		9 (13.85)	0	
AFP level, N (%)^b^			0.416			0.001
< 200 ng/ml	13 (41.94)	35 (50.72)		23 (35.38)	25 (71.43)	
≥ 200 ng/ml	18 (58.06)	34 (49.28)		42 (64.62)	10 (28.57)	

*SD* Standard deviation, *IQR* Interquartile range, *HCV* Hepatitis C virus, *HBV* Hepatitis B virus, *AST* Aspartate aminotransferase, *ALT* Alanine transaminase, *INR* International normalised ratio, *CRP* C-reactive protein, *BCLC* Barcelona Clinic Liver Cancer, *AFP* Alpha-fetoprotein. ^a^Variables that were significantly different between patients with and without sarcopenia. ^b^Variables that were significantly different between IMF and non-IMF patients

### Association between IMF deposition and HCC survival

As shown in Fig. 1, patients with low MA and IMF deposition had a significantly shorter median survival than those with high MA (62.0 ± 13.82 versus 457 ± 77.13 days, *P* < 0.0001). In addition, the low MA group had a higher mortality rate (HR, 3.580; 95% CI, 1.895–6.764; *P* < 0.001) in multivariate analysis (Table 2), suggesting that IMF deposition is associated with the progression of liver disease. An MA value ≤44.4 HU for men or ≤ 39.3 HU for women was established as the threshold for IMF deposition, and there was no significant difference between the proportion of men and women with a low MA value (Table 3).

As summarised in Table 3, patients with IMF deposition had significantly higher AST and CRP levels, which indicate the presence of local and systemic inflammation. In addition, the IMF deposition group showed higher AFP, INR, and CP score, and lower sodium and albumin levels, which are markers of the severity of liver cancer. In subgroup analyses, IMF deposition was significantly associated with poor survival among patients of the same gender (male, log-rank *P* < 0.0001), same age group (age ≥ 55 years old, log-rank *P* < 0.0001), same BCLC stage (BCLC stage C, log-rank *P* = 0.0004) and same JIS score (JIS score 3, log-rank *P* = 0.0006). This suggested that IMF deposition is a robust predictor of HCC survival (Fig. 2).

### HCC staging system and significant factors affecting survival

**Fig. 3:**
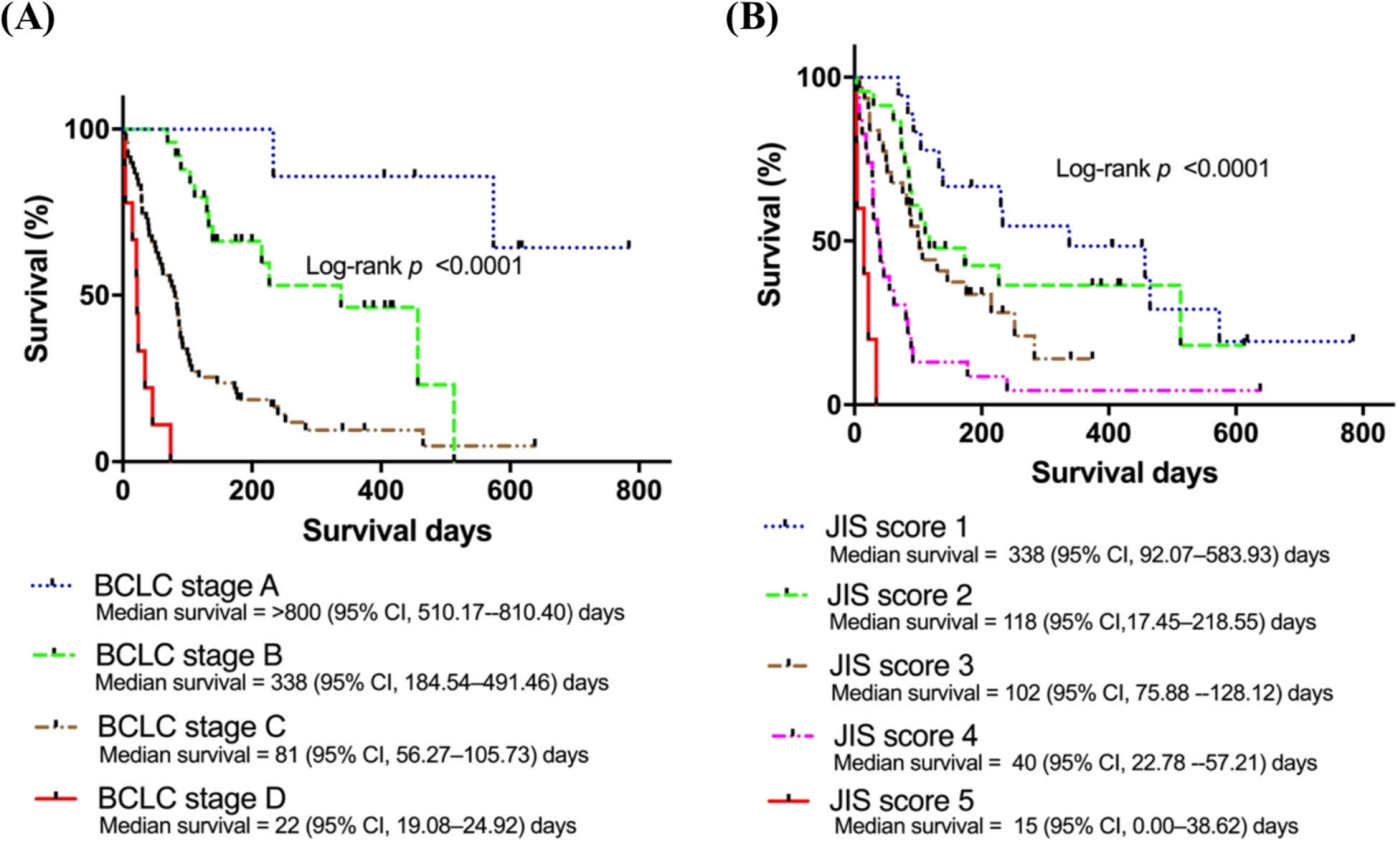
Staging systems and prognosis of HCC. Higher BCLC stage (**a**) and JIS score (**b**) were significantly associated with lower survival on Kaplan–Meier analysis. BCLC, Barcelona Clinic Liver Cancer; CI, confidence interval; JIS, Japan Integrated Staging

To identify the optimal staging system for predicting prognosis and survival in Indonesian patients, we evaluated methods based on the anatomical characteristics of the tumour (TNM classification) and methods that combine the anatomical features of the tumour with an integrated assessment of liver disease, such as the BCLC stage and JIS score. As depicted in Fig. 3, BCLC stage and the JIS score were significantly associated with poor survival in the Kaplan–Meier analysis, showing superior predictive power. These two systems were identified as independent predictors of survival compared with other prognostic systems (LCSGJ TNM stage and AJCC TNM stage 8th edition) as shown in Table 2.

Univariate Cox analysis showed that the factors significantly associated with increased mortality were gender (*P* = 0.029; HR, 1.845), sarcopenia (*P* < 0.001; HR, 2.950), IMF deposition (*P* < 0.001; HR, 5.487), HBV infection (*P* = 0.002; HR, 2.326), CP class (*P* < 0.001; HR, 5.069 for class C), LCSGJ TNM stage (*P* < 0.001; HR, 3.308 for stage 4), AJCC 8th ed. TNM stage (*P* < 0.001; HR, 3.663 for stage 4), JIS score (*P* < 0.001; HR, 28.373 for score 5), BCLC stage (*P* < 0.001; HR, 65.958 for stage D), and AFP ≥200 ng/ml (*P* = 0.006; HR, 1.916). Multivariate analysis showed that gender (*P* < 0.001; HR, 3.211), sarcopenia (*P* = 0.016; HR, 1.921), IMF deposition (*P* < 0.001; HR, 3.580), JIS score (*P* = 0.020; HR, 2.067 for score 4), and BCLC stage (*P* = 0.003; HR, 6.131 for stage D) were independently and significantly associated with reduced survival. The detailed results of the univariate and multivariate analyses of liver-related mortality are shown in Table 2.

## Discussion

To the best of our knowledge, this is the first study to show that body composition measures are associated with the prognosis of HCC in Indonesian patients. This study showed that both sarcopenia and IMF deposition predicted poor survival of HCC patients independently of demographics, cancer stage, and the degree of liver dysfunction.

In a large-scale retrospective cohort study of 1257 Japanese patients, both quantity (defined by low SMI) and quality (determined by low MA) of muscle were identified as significant predictors of HCC survival [2]. In Japan, where a national surveillance program has been applied to the high-risk population since 1980, 62% of HCC cases are diagnosed at BCLC stage A, and the median survival time for HCC patients is 47.2 months, which is considered the best in the world [6, 11]. In Japan, HCC is mostly caused by hepatitis C virus infection and is more prevalent in the older population (mean age, 68 years); this may affect sarcopenia status, as this term also refers to the age-related decline in muscle mass and function [2]. However, in Indonesia, the prevalent characteristics related to HCC are advanced-stage presentation, early-age onset, and HBV endemicity, which differ from those in Japan [6]. The present findings suggest that sarcopenia and IMF deposition could be used to predict the outcomes of HCC patients regardless of age, the causative virus, and stage at diagnosis.

Sarcopenia, derived from the Greek words “sarcos” for flesh and “penia” for deficiency, was initially used to describe the decline in muscle mass and function related to the aging process [12]. However, later studies examined sarcopenia occurring in association with several types of cancer, such as ovarian cancer, lung cancer, pancreatic cancer, renal cell carcinoma, oesophageal cancer, and lymphoma, as this condition affects the overall survival of patients [9, 13]. Sarcopenia is also correlated with poor quality of life (QOL) and symptoms of depression, cognitive impairment, inflammatory bowel disease, and chronic kidney disease [14–16].

Regarding liver disease, studies show that sarcopenia is associated with poor outcomes in patients with cirrhosis and HCC with reduced tolerance to chemotherapy [2, 17–19]. Recent studies show that sarcopenia is associated with low-grade systemic inflammation, as indicated by increased inflammatory cytokines (IL-1, IL-6, and TNFα) leading to oxidative stress, which together with mitochondrial dysfunction, could be central to the pathogenesis of sarcopenia [2, 10, 20]. Inflammation and stress-related signalling pathways, including nuclear factor-κB and signal transducer and activator of transcription, are critical players in the progression of liver fibrosis and HCC development [21], which could explain the association of sarcopenia with poor prognosis in our study (Fig. 1).

In addition to the decline of muscle mass, adipose tissue deposition in skeletal muscle has recently attracted interest in cancer research [2, 10, 13]. Normal skeletal muscles contain a small amount of fat that is used as a source of energy during aerobic activity [22]. An increase in muscle lipid content associated with decreased CT attenuation values within the muscle may represent a marker of myosteatosis or a possible surrogate for muscle function and quality [22, 23].

Low average skeletal muscle attenuation on CT is a more important indicator of a patient’s functional status than the quantification of muscle mass [13]. The degree of adipose infiltration into skeletal muscle, the liver, and other organs is associated with increased triglyceride content and reduced insulin-stimulated glucose uptake, which interferes with insulin signalling and leads to insulin resistance [10, 23, 24]. Low skeletal muscle attenuation is associated with deficits in physical function, altered metabolism, and poor prognosis [23]. A previous study showed that muscle adiposity is strongly correlated with inflammation, which may depend on the secretory profile of adipocyte-derived factors, as shown by increased IL-6 expression and CRP levels in patients with intramuscular fat deposition [10].

In the present study, HCC patients with IMF deposition had significantly higher levels of AST and CRP, indicative of local and systemic inflammation (Table 3). Further basic and clinical research focusing on intramuscular adipocytes and myosteatosis is warranted in the HCC population, particularly given the unique set of metabolic derangements associated with the disease.

The present results suggest that BCLC stage and the JIS score are superior indicators for predicting patient outcomes in HCC (Table 2). The functional impairment caused by the underlying liver disease has a significant impact on outcomes, irrespective of tumour stage [8]. For this reason, systems that include only the anatomical characteristics of the tumour, such as the AJCC and LCSGJ staging systems, which stratify patients using the TNM classification, do not have an excellent predictive capability [8].

Analyses performed in US and Asian patients of any stage demonstrated that the BCLC system has a stronger predictive power than other systems, since it integrates information about tumour extension, liver function, and the presence of constitutional symptoms, providing important information to guide therapeutic choices [8, 25, 26]. The JIS score, which combines TNM and Child–Pugh and is widely used in Japan, showed good predictive value regarding survival in Indonesian HCC patients in our study [27].

In the present study, survival was worse in men than in women (Table 2). The gender disparity of HCC is an essential topic in hepatocarcinogenesis, as the incidence and mortality of HCC are significantly lower in women than in men [28]. Postmenopausal women are at a higher risk of developing HCC, suggesting that oestrogen has protective effects on HCC development and progression [28]. The male predominance of HCC could also be associated with androgen and androgen receptor (AR) signalling [29]. Increased testosterone levels are significantly associated with the risk of HCC. Androgen/AR signalling may promote early-stage hepatocarcinogenesis by increasing cell growth through the transcriptional regulation of transforming growth factor beta 1 and the modulation of cell cycle-related kinase transcription [29]. However, a study of HCC survival conducted in Japan showed no significant difference in survival between men and women [2]. Further investigation of the roles of hormones in HCC progression is warranted, particularly because the efficacy of hormone therapy for liver cancer remains controversial [30].

The present findings support that sarcopenia and IMF deposition are independent predictors of the survival of HCC patients, as shown in several studies in other populations [2, 17–19]. We believe that measurement of skeletal muscle mass and quantity should be added to widely used staging systems for HCC stratification in clinical practice. The present study had some limitations related to the small sample size and limited duration of observation. We attempted to overcome these challenges by performing a prospective cohort study in which patient status was followed-up each month to determine the correlation between skeletal muscle measurement and mortality. However, we were unable to address the question of causality in the present study. Additional basic research and larger clinical studies are necessary to clarify these issues.

Other limitation of our study is that we used a definition of sarcopenia based on cut-off values from CT measurements only and did not assess muscle function (strength or performance) for the diagnosis of sarcopenia. However, as also applied by other studies, imaging tools were used for evaluating and diagnosing sarcopenia in the clinical and research settings [9, 13]. In addition, we only included patients enrolled in Yogyakarta Province Hospital (RSUP Dr. Sardjito), a tertiary hospital that receives patients referred from several provinces in Indonesia. Furthermore, although the patients included were mostly Javanese, it is the most prevalent ethnic population in Indonesia (40.06%) [5]. Therefore, we believe that our data are representative of the overall Indonesian population.

## Conclusions

In conclusion, we showed that skeletal muscle quantity and quality measurements can predict the outcomes of Indonesian patients with HCC. These factors may be linked to prognosis through various mechanisms, such as increased inflammation levels leading to disease progression. We also showed that BCLC stage and the JIS score had superior predictive value over other staging systems for predicting survival among Indonesian HCC patients. Increased awareness and early management strategies to prevent or reverse sarcopenia and IMF deposition in HCC patients might improve survival rates and outcomes.

## Data Availability

All data and material in published, mentioned and referenced studies are available from the corresponding author on reasonable request.

## Abbreviations

AFP: Alpha-fetoprotein
AST: Aspartate aminotransferase
BCLC: Barcelona clinic liver cancer
BMI: Body mass index
CP: Child–turcotte–pugh
CRP: C-reactive protein
CT: Computed tomography
HBV: Hepatitis B virus
HCC: Hepatocellular carcinoma
HCV: Hepatitis C virus
IMF: Intramuscular fat
INR: International normalised ratio
JIS: Japan integrated staging
LCSGJ: Liver cancer study group of japan
MA: Muscle attenuation
QOL: Quality of life
RFA: Radiofrequency ablation
SMI: Skeletal muscle index
TACE: Transarterial chemoembolization
TNM: Tumour node metastasis
